# Endoscopic Diagnosis of *Necator americanus* Infection Presenting With Persistent Iron‐Deficiency Anemia: Usefulness of Image‐Enhanced Endoscopy and Capsule Endoscopy

**DOI:** 10.1002/deo2.70352

**Published:** 2026-05-22

**Authors:** Tomohiro Takebe, Satoshi Osawa, Yukihiro Watanabe, Shingo Yamagata, Takuya Aoyama, Yusuke Kawamura, Airi Manabe, Taro Kisamori, Munetaka Sano, Ken Sugimoto

**Affiliations:** ^1^ First Department of Medicine Hamamatsu University School of Medicine Shizuoka Japan; ^2^ Department of Gastroenterology Yaizu City Hospital Shizuoka Japan; ^3^ Department of Advanced Medical Science for Regional Collaboration Hamamatsu University School of Medicine Shizuoka Japan; ^4^ Department of Endoscopic and Photodynamic Medicine Hamamatsu University School of Medicine Shizuoka Japan

**Keywords:** capsule endoscopy, Hookworm infection, iron‐deficiency anemia, narrow‐band imaging, *Necator americanus*

## Abstract

*Necator americanus* infection is now rare in developed countries but remains an important cause of iron‐deficiency anemia and abdominal symptoms in individuals with relevant epidemiological backgrounds. A 59‐year‐old Filipino man with long‐standing unexplained iron‐deficiency anemia was admitted to our hospital for epigastric pain associated with choledocholithiasis. Although biliary enzymes improved after endoscopic treatment, his abdominal pain persisted. Subsequent gastrointestinal investigations, including upper gastrointestinal endoscopy, colonoscopy, and small‐bowel capsule endoscopy, revealed multiple parasites distributed throughout the gastrointestinal tract. Image‐enhanced endoscopy using narrow‐band imaging clearly improved visualization of the parasites compared with conventional white‐light imaging, facilitating their identification and endoscopic removal. Genetic analysis of the extracted worms confirmed *N. americanus* infection. The patient was treated with pyrantel pamoate, which resulted in the resolution of abdominal symptoms and improvement of iron‐deficiency anemia. Follow‐up stool examination confirmed eradication of the parasite. This case highlights the importance of considering hookworm infection in patients with persistent iron‐deficiency anemia and abdominal pain, even in developed countries. In addition, image‐enhanced endoscopy and capsule endoscopy are valuable diagnostic tools for detecting hookworms and assessing their distribution within the gastrointestinal tract.

## 1 Introduction

*Necator americanus* is a hookworm species that attaches to the mucosa of the small intestine and feeds on host blood, leading to iron‐deficiency anemia and gastrointestinal symptoms [1, 2]. Although hookworm infection was once endemic in Japan, its prevalence has markedly declined with improvements in sanitation and living conditions. Currently, most cases encountered in developed countries are associated with travel or migration from endemic regions [3].

Because adult hookworms primarily inhabit the proximal small intestine, diagnosis can be difficult using standard endoscopic examinations alone [4]. Recent advances in endoscopic imaging and capsule endoscopy have enabled more detailed visualization of parasites within the gastrointestinal tract [5]. Here, we report a rare case of *N. americanus* infection presenting with persistent IDA, in which image‐enhanced endoscopy and capsule endoscopy played key diagnostic roles.

## 2 Case Report

A 59‐year‐old Filipino man was followed at our hospital for unexplained iron‐deficiency anemia. Previous upper gastrointestinal endoscopy showed no abnormalities, and fecal occult blood tests were negative. He had been treated with oral iron supplementation without definitive improvement.

Five days before admission, he developed epigastric pain and visited a local clinic. Laboratory tests revealed elevated biliary enzymes, and abdominal computed tomography demonstrated gallstones and suggested a small (approximately 2 mm) calcified lesion in the distal common bile duct, without significant bile duct dilation. He was referred to our hospital and admitted with suspected choledocholithiasis.

His medical history included pulmonary tuberculosis at the age of 52 years. He had no significant family history and was not taking any regular medications. On admission, his height was 166 cm, and his body weight was 54 kg. His body temperature was 37.2 °C, blood pressure 134/91 mmHg, and pulse rate 90 beats/min. Abdominal examination revealed mild epigastric tenderness without rebound tenderness.

Laboratory findings showed hemoglobin 10.0 g/dL, serum iron 21 µg/dL, total iron‐binding capacity 333 µg/dL, and ferritin 24.3 ng/mL, consistent with iron‐deficiency anemia. The eosinophil count was 454/µL (5.9%), and total protein and albumin levels were 6.9 and 3.2 g/dL, respectively. Liver and biliary enzymes were elevated, and C‐reactive protein was 5.75 mg/dL.

Endoscopic retrograde cholangiopancreatography (ERCP) was performed for suspected cholangitis due to choledocholithiasis. Although the stone was not clearly visualized during ERCP, endoscopic sphincterotomy and temporary biliary stent placement were performed based on the clinical suspicion of choledocholithiasis. Although biliary enzymes improved, the patient's abdominal pain persisted, prompting further investigation.

**FIGURE 1:**
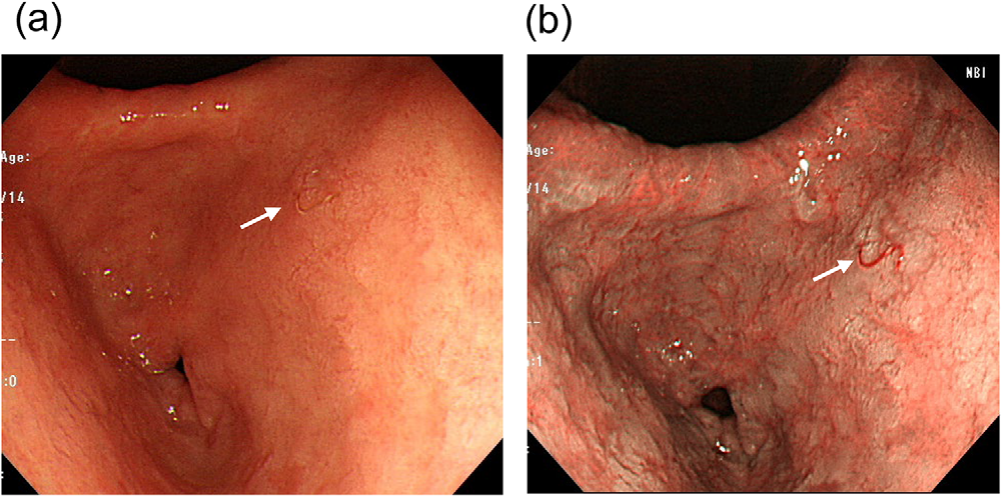
Upper gastrointestinal endoscopy findings. (a) White‐light imaging shows a hookworm in the gastric antrum. The arrow indicates the parasite. (b) Narrow‐band imaging enhances the contrast between the hookworm and the surrounding mucosa, allowing clearer visualization of the parasite. The arrow indicates the parasite.

**FIGURE 2:**
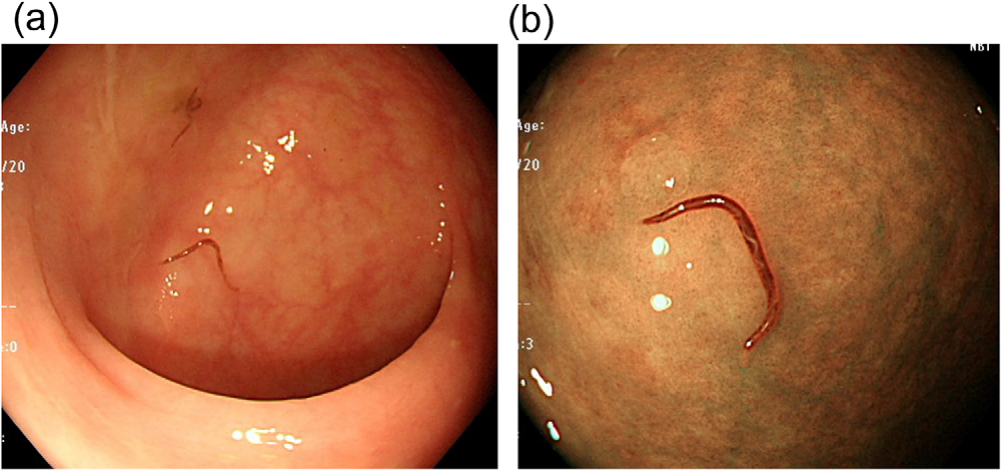
Colonoscopy findings. (a) White‐light imaging demonstrates hookworms in the terminal ileum and colon. (b) Narrow‐band imaging markedly improves visualization of the worm body and attachment site. The head of the approximately 20 mm string‐like body penetrates the mucous membrane and sucks blood.

Upper gastrointestinal endoscopy revealed single parasites in the gastric antrum and duodenal bulb. Narrow‐band imaging (NBI) clearly enhanced the visualization of the parasites compared with white‐light imaging, especially in the stomach (Figure 1a,b). Colonoscopy demonstrated multiple parasites from the terminal ileum to the entire colon, without significant mucosal inflammation, erosions, or ulcers (Figure 2a). NBI clearly enhanced visualization of the parasites compared with white‐light imaging, facilitating identification and endoscopic removal using biopsy forceps (Figure 2b).

**FIGURE 3:**
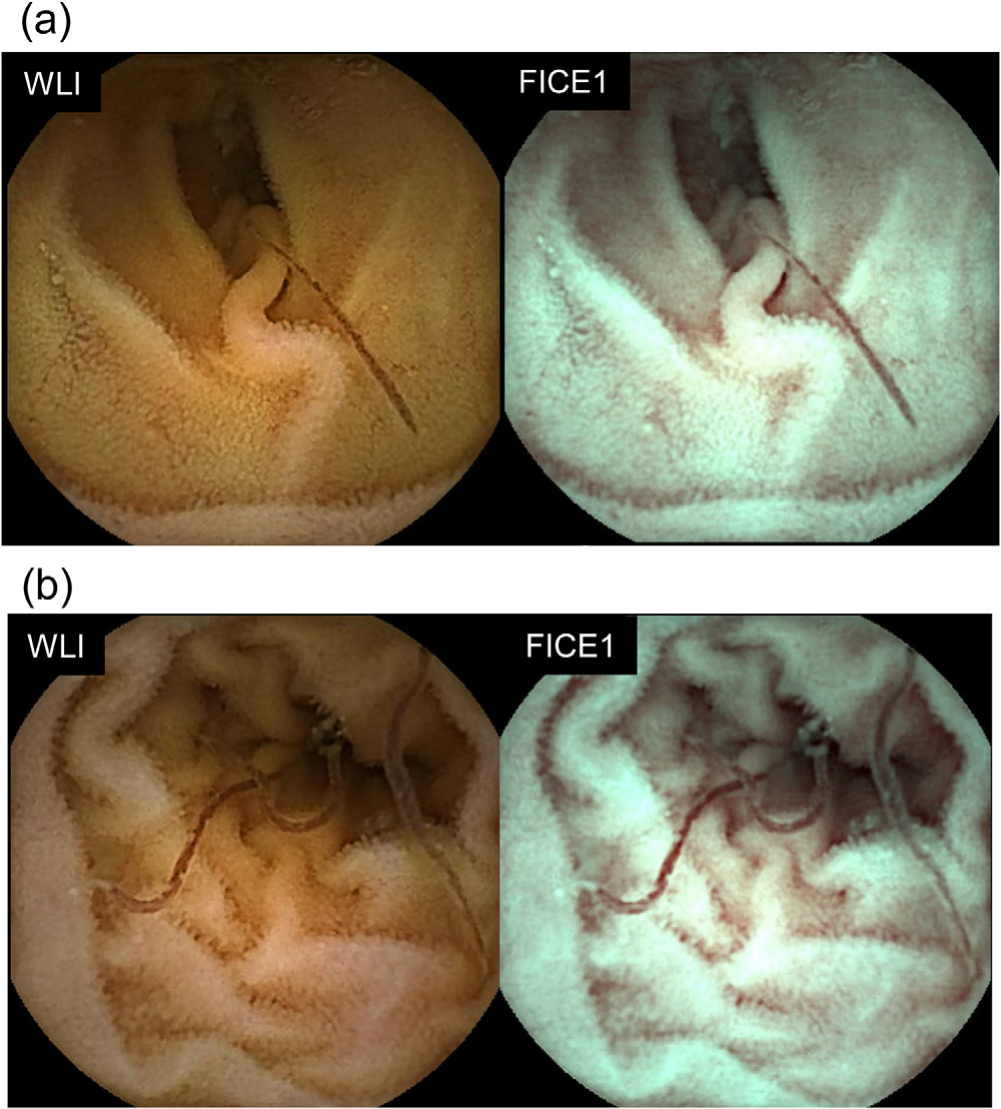
Small‐bowel capsule endoscopy findings. Multiple hookworms are observed attached to the small‐intestinal mucosa at various sites. (a) Image of the Jejunum. (b) Image of the ileum. Left: White‐light imaging. Right: Image‐enhanced observation using flexible spectral imaging color enhancement (FICE1 mode). Using the FICE1 mode improves the visibility of the hookworms.

Small‐bowel capsule endoscopy was performed to confirm the distribution of parasites within the small intestine. As shown in Figure 3, parasites were identified throughout the small intestine from the jejunum to the ileum, and FICE1 images also improved the visibility of the parasites, similar to the effect observed with NBI (Figure 3 and Video S1).

Extracted parasites by colonoscopy were sent to the National Institute of Infectious Diseases, where genetic analysis confirmed female *N. americanus*. Genetic identification was performed by polymerase chain reaction targeting the internal transcribed spacer (ITS) region of ribosomal DNA, encompassing ITS1, 5.8S, and ITS2. The obtained sequence (980 bp) showed 99.9%–100% identity with reference *N. americanus* sequences in GenBank (e.g., Accession Nos. LC036565, PX590269, and LC036563), confirming the species. The patient was treated with pyrantel pamoate (500 mg/day for three consecutive days). After deworming, his abdominal symptoms resolved, and his iron‐deficiency anemia improved with iron supplementation. Follow‐up stool examination one month later and colonoscopy 9 months later confirmed eradication of the parasite, and no recurrence involving iron‐deficiency anemia has been observed at 1‐year follow‐up after treatment.

## 3 Discussion

Hookworm infection is caused mainly by *N. americanus* and *Ancylostoma duodenale*, both of which use humans as their definitive hosts [1]. *N. americanus* is widely distributed in tropical and subtropical regions, particularly in Southeast Asia, sub‐Saharan Africa, and Latin America. Infection occurs mainly through percutaneous penetration of filariform larvae from contaminated soil. Typical clinical manifestations include chronic iron‐deficiency anemia, abdominal discomfort, and occasionally gastrointestinal bleeding in cases of heavy infestation [6]. Our patient had previously worked barefoot as a farmer in the Philippines, strongly suggesting percutaneous infection before migration.

Adult *N. americanus* feeds on host blood, with an estimated blood loss of approximately 0.03 mL per worm per day [1]. Heavy infestation can therefore result in chronic iron‐deficiency anemia. Although historically referred to as “duodenal hookworm,” adult worms are more commonly found in the proximal jejunum than in the duodenum. This distribution may explain why conventional upper gastrointestinal endoscopy alone often fails to detect the parasites [4].

In fact, this patient had also undergone upper gastrointestinal endoscopy 1 year prior, but no parasites were found at that time. During the subsequent diagnostic workup, in addition to upper and lower endoscopy, small‐bowel capsule endoscopy was also performed, leading to the diagnosis of parasites. In the present case, parasites were detected not only in the duodenum but also in the stomach, colon, and throughout the small intestine. Gastric infestation by *N. americanus* is considered rare because the parasite is vulnerable to gastric acid [7]. Possible explanations include temporary movement due to peristalsis or changes in the gastric acid environment due to the use of gastric acid suppressants during the treatment of choledocholithiasis, but the exact mechanism remains unclear.

An important feature of this case is the diagnostic contribution of image‐enhanced endoscopy. NBI enhanced visualization of the parasites because hookworms containing ingested blood strongly absorb the specific wavelengths used in NBI, creating higher contrast against the surrounding non‐vascular mucosa [8, 9]. This facilitated prompt recognition and endoscopic removal. Furthermore, capsule endoscopy using FICE1 mode enabled comprehensive assessment of parasite distribution in the small intestine, which has rarely been reported in previous cases [3].

Benzimidazole derivatives such as albendazole and mebendazole are generally recommended as first‐line treatments for hookworm infections [1], but pyrantel pamoate is also an effective alternative [10]. In the present case, a three‐day regimen of 500 mg pyrantel pamoate, which is readily available commercially in Japan, resulted in complete clinical and parasitological resolution. Although stool examination for ova is widely used as a screening method, it should be noted that its sensitivity may be limited, particularly in cases with low worm burden or intermittent egg shedding [5].

This case underscores the importance of considering hookworm infection in patients with unexplained iron‐deficiency anemia and abdominal symptoms, particularly in those with epidemiological risk factors. In such cases, careful endoscopic observation, including the use of image‐enhanced modalities and capsule endoscopy, may be crucial for diagnosis.

In conclusion, we report a rare case of *N. americanus* infection diagnosed by gastrointestinal endoscopy in a developed country. Image‐enhanced endoscopy and capsule endoscopy were particularly useful for detecting and characterizing the parasites. Hookworm infection should be included in the differential diagnosis of persistent iron‐deficiency anemia and abdominal pain in patients with relevant travel or migration histories.

## Author Contributions

**Shingo Yamagata**: investigation and data curation. **Tomohiro Takebe**: conceptualization, writing – original draft, data curation, and investigation. **Yukihiro Watanabe**: investigation and data curation. **Airi Manabe**: investigation and data curation. **Ken Sugimoto**: writing – review and editing and supervision. **Takuya Aoyama**: data curation and investigation. **Munetaka Sano**: conceptualization and writing – review and editing. **Yusuke Kawamura**: investigation and data curation. **Taro Kisamori**: investigation and data curation.

## Conflicts of Interest

The authors declare no conflicts of interest.

## Funding

The authors have nothing to report.

## Ethics Statement

The patient in this case report was treated within the standard scope of care under Japan's national health insurance. Informed consent was obtained from the patient for the medical treatment and procedures described. In publication, all accompanying images and videos have been anonymized to ensure that the individual cannot be identified.

## Consent

The patients provided informed consent for the publication of this case report.

## Supporting information




**Supporting VIDEO S1**: Small‐bowel capsule endoscopy revealed that multiple string‐like hookworms, approximately 20 mm in length, were present throughout the small intestine, partially attached to the mucosa.

## References

[1] P. J. Hotez , S. Brooker , J. M. Bethony , M. E. Bottazzi , A. Loukas , and S. Xiao , “Hookworm Infection,” New England Journal of Medicine 351, no. 8 (2004): 799–807. 15317893 10.1056/NEJMra032492

[2] S. N. Tiremo and M. S. Shibeshi , “Endoscopic Diagnosis of Hookworm Disease in a Patient With Severe Iron Deficiency Anemia: A Case Report,” International Medical Case Reports Journal 16 (2023): 841–845. 38116465 10.2147/IMCRJ.S443625 PMC10729675

[3] U. C. Ghoshal , A. Venkitaramanan , A. Verma , A. Misra , and V. A. Saraswat , “Hookworm Infestation Is Not an Uncommon Cause of Obscure Occult and Overt Gastrointestinal Bleeding in an Endemic Area: A Study Using Capsule Endoscopy,” Indian Journal of Gastroenterology 34, no. 6 (2015): 463–467. 26631236 10.1007/s12664-015-0611-2

[4] B. C. Sharma , D. K. Bhasin , H. S. Bhatti , G. Das , and K. Singh , “Gastrointestinal Bleeding due to Worm Infestation, With Negative Upper Gastrointestinal Endoscopy Findings: Impact of Enteroscopy,” Endoscopy 32, no. 4 (2000): 314–316. 10774972 10.1055/s-2000-7393

[5] X. Tan , M. Cheng , J. Zhang , et al., “Hookworm Infection Caused Acute Intestinal Bleeding Diagnosed by Capsule: A Case Report and Literature Review,” Korean Journal of Parasitology 55, no. 4 (2017): 417–420. 28877573 10.3347/kjp.2017.55.4.417 PMC5594724

[6] J. Bethony , S. Brooker , M. Albonico , et al., “Soil‐transmitted Helminth Infections: Ascariasis, Trichuriasis, and Hookworm,” Lancet 367, no. 9521 (2006): 1521–1532. 16679166 10.1016/S0140-6736(06)68653-4

[7] A. Dumont , V. Seferian , and P. Barbier , “Endoscopic Discovery and Capture of Necator Americanus in the Stomach,” Endoscopy 15, no. 2 (1983): 65–66. 6851955 10.1055/s-2007-1021467

[8] Image of the Month: Hookworm Infection Observed via a Narrow‐band Imaging System. American Journal of Gastroenterology 2014, 109, no. (5):626. 10.1038/ajg.2014.96 24797001

[9] S. S. Rana , R. Sharma , V. Sharma , and R. Gupta , “Blood Sucking Hookworm Under Narrow Band Imaging,” Digestive and Liver Disease 49, no. 9 (2017): 1058. 28462888 10.1016/j.dld.2017.03.024

[10] M. Ahmed , “Intestinal Parasitic Infections in 2023,” Gastroenterology Research 16, no. 3 (2023): 127–140. 37351081 10.14740/gr1622 PMC10284646

